# Procalcitonin as prognostic marker of mortality

**DOI:** 10.1186/cc11967

**Published:** 2013-03-19

**Authors:** S Zampieri, P Bettonte, M Ortolani, G Frison, V Schweiger, L Gottin, E Polati

**Affiliations:** 1 Policlinico G.B. Rossi, Verona, Italy; 2 Santa Chiara Hospital, Trento, Italy; 3 Ospedale Maggiore Civile, Verona, Italy

## Introduction

We analyze procalcitonin (PCT) as a prognostic marker, in order to assess the clinical impact of a daily PCT measure.

## Methods

From November 2010 to November 2011 we collected clinical data, drug administration, scores and PCT values of 420 consecutive patients during hospitalization. Statistical analysis was made using SPSS software. We calculated ICU mortality, 1-month mortality and 1-year mortality. Median percentage daily variation was calculated as: (PCT day after - PCT of the date value)/PCT of the date value×100. PCT variation in the last 48 hours of hospitalization was calculated as: (PCT at discharge - PCT at 48 hours before discharge)/PCT 48 hours before discharge×100. We compared peak values in dead patients versus alive patients. A logistic regression was performed in order to assess mortality odds ratio.

## Results

Of the 420 patients, 63 (15%) died in the ICU, 12 (2.86%) died 1 month after ICU discharge and 16 (3.80%) died 1 year after ICU discharge. PCT values were higher during the last day of hospitalization in dead patients versus alive patients. PCT percentage variation during the last 48 hours of hospitalization had a slower trend in patients who died than in those who survived; these differences are even more marked in patients who had a septic event. A slower descending trend of daily PCT values was found in patients who died than in those who survived. PCT peak levels during the ICU stay were higher in dead patients with respect to alive ones. At logistic regression analysis PCT decrease in the last 48 hours <-30% (OR 3.71), PCT peak higher than 10 ng/ml (OR 2.38), and PCT last day/PCT peak ratio >50% (OR 2.064) were ICU mortality risk factors. PCT values were a higher predictive ICU mortality risk factor than SOFA and APACHE II scores. Other prognostic factors were age and lactate values. Only age was a risk factor in 1-month and 1-year mortality.

## Conclusion

PCT is a good prognostic marker and is strongly correlated to the clinical status and gravity of the patients, so PCT seems to be a useful marker in an intensive care scenario.
